# Gradient Titanium Alloy with Bioactive Hydroxyapatite Porous Structures for Potential Biomedical Applications

**DOI:** 10.3390/ma17225511

**Published:** 2024-11-12

**Authors:** Julia Sadlik, Edyta Kosińska, Magdalena Bańkosz, Agnieszka Tomala, Grzegorz Bruzda, Josef Jampilek, Agnieszka Sobczak-Kupiec

**Affiliations:** 1 Cracow University of Technology, CUT Doctoral School, Faculty of Materials Engineering and Physics, Department of Materials Science, Faculty of Materials Engineering and Physics, Cracow University of Technology, 37 Jana Pawła II Av., 31-864 Krakow, Poland; edyta.kosinska@doktorant.pk.edu.pl (E.K.); magdalena.bankosz@pk.edu.pl (M.B.); 2 Cracow University of Technology, Faculty of Materials Engineering and Physics, Department of Materials Science, 37 Jana Pawła II Av., 31-864 Krakow, Poland; agnieszka.tomala@pk.edu.pl (A.T.); agnieszka.sobczak-kupiec@pk.edu.pl (A.S.-K.); 3 Łukasiewicz Research Network—Krakow Institute of Technology, Zakopiańska 73 Str., 30-418 Krakow, Poland; grzegorz.bruzda@kit.lukasiewicz.gov.pl; 4 Department of Analytical Chemistry, Faculty of Natural Sciences, Comenius University, Ilkovicova 6, 842 15 Bratislava, Slovakia; 5 Department of Chemical Biology, Faculty of Science, Palacky University Olomouc, Slechtitelu 27, 783 71 Olomouc, Czech Republic

**Keywords:** hydroxyapatite, titanium alloys, Ti6Al4V, biomaterials, porous materials

## Abstract

Hard bone disease is a clinical problem affecting more than 20 million people annually worldwide, with significant health, social, and economic consequences. For successful integration of any implant, the key aspects are bone regeneration, osseointegration at the bone–implant interface, and the mitigation of inflammation. The purpose of this research work is to demonstrate an innovative material system and method of biomaterial preparation for regenerative medicine. A number of studies were carried out for both hydroxyapatite powder and composites. Wet-precipitated synthesized hydroxyapatite was compared to commercial products through accurate physicochemical studies that confirmed the high purity of the obtained calcium phosphate without any impurities. Ti/HAp composites before and after sintering were compared by XRF, XRD, SEM, EDS, PSA, and roughness measurements, and the Vickers microhardness was analyzed. The fabrication of the biomaterial was based on a bottom-up approach, which involved fabricating HAp particles with specific morphologies using powder metallurgy (PM) to sinter Ti composites. The resulting gradient structures consisting of two compositions (5%HAp%5CMC and 10%HAp10%CMC) mimic the structure of bone tissue. The created pores of 10–100 µm in size will allow bone cells to penetrate the implant and regenerate bone. In turn, the introduction of hydroxyapatite into the material reduces the microhardness of the composite and introduces properties such as bioactivity. The developed composite material contains a combination of Ti alloy and hydroxyapatite (HAp), creating an excellent biomaterial that promotes bone growth and eliminates the problem of implant loosening by integrating it into the bone. This material requires further research, especially biological research. However, it shows promising potential for further experiments.

## 1. Introduction

Current biomaterials are required to exhibit good biological compatibility (biostability or biodegradability), appropriate surface morphology, good mechanical properties, (low density, high strength), and tribological properties (resistance to fatigue, abrasion, and corrosion) [1,2]. In addition to biological and mechanical compatibility, the final product should be relatively easy to produce and have good processability, including joining and casting [3]. Current efforts are focused on the production of biomaterials, including cellular structures like specified porosity, supporting the regeneration of damaged tissues by the ingrowth of cells into the implant surface, and mechanical and chemical surface treatments like laser texturing, introducing bioactive particles in the matrix or covering the implant surface with a coating [4,5,6,7]. Material selection should be determined for critical bone size defects by load-bearing implants [8]; e.g., to replace tissue in the more loaded regions like the knee or hip, pure metals and their alloys are recommended. However, it often appears the various parts of the implant operate under different conditions. In order to obtain the best results, it is, therefore, understandable that advanced implants are immediately often produced with ceramic, plastic, and metallic materials [9].

The most commonly used metallic materials in implant production are titanium and its alloys [10]. Titanium alloys are very stable in biological environments such as bodily fluids and do not corrode. Additionally, titanium has been proven to support cell growth and differentiation, and it readily absorbs proteins from the body’s fluid [11,12]. The good biocompatibility of titanium materials is owing to the spontaneously formed strong and stable oxide layer on its surface, providing corrosion resistance [13]. There are three groups of titanium useful in the biomedical field: commercial pure titanium (CP), α + β alloys, and β alloys. The first titanium alloy used for biomedical applications was Ti-6Al-4V [14].

Despite the widespread use of Ti for implants, its bio-inertness limits its use for applications where the Ti part has to be fixed to the bone host. An insufficient bond formation between the bone and implant surface would cause micro-movements, leading eventually to a loosening of the implant and, finally, to its failure by cracking [15]. To enhance the bioactivity and achieve proper bone-on-bone bonding, the Ti matrix can be reinforced with ceramic particles using incremental manufacturing and powder metallurgy techniques [16]. The use of incremental techniques in reactive gas atmospheres is also becoming increasingly popular. The Ti matrix can be strengthened in situ by applying nitrogen. This method results in improved properties of pure metals but also of alloys [17]. It is well known that bioactive ceramics support osseointegration. By covering the Ti surface with a bioactive coating, the adhesion of the implant to the bone is enhanced, thus resulting in significantly better implant lifetimes [18,19].

Hydroxyapatite (HAp, Ca_10_(PO_4_)_6_(OH)_2_) is one of the best-known ceramic materials used in the field of tissue engineering as a surface coating as well as for reinforcing particles [20]. HAp, due to its well-known biocompatibility, osteoconductive and osteoinductive properties, and tendency to form a strong bond to bone tissue, has become widely used in orthopedic and dental applications, including uses such as bone and tooth cavity filling, small and unloaded implants, porous scaffolds for cell in-growth, and bioactive coatings on load-bearing metal implants and the bioactive phase in a polymer matrix biocomposite [21,22,23]. HAp is an ideal mineral substitute in view of its chemical composition to the biomineral in the hard tissues [24]. Moreover, it has been proved that Hap-based materials easily combine with newly formed bone and promote the regeneration of damaged bone tissue [25]. Studies have demonstrated the stimulating effect of hydroxyapatite for bone reconstruction, due to a process of resorption in the human body, which allows the use of this material as a scaffold or carrier for reconstructing bone tissue [26]. However, its mechanical properties, such as low fracture toughness, poor tensile strength, and weak wear resistance, make it insufficient as a load-carrying material [27]. The mentioned problem can be solved by producing composite biomaterials obtained by conventional powder metallurgy (PM) techniques, which allow us to create superior mechanical and biological properties without any mechanically weak interface. Moreover, during the PM process, the pore morphology and porosity can be precisely controlled by the pressing and sintering parameters as well as the addition of porophore. By tuning these parameters, a good supply of body fluids for growing bone cells, the acceleration of CaP phase dissolution and reprecipitation processes on the implant surface, and subsequent tissue bonding can be ensured, which finally enhances the bone formation on the implants’ porous surface.

The scope of this work starts with the synthesis of bioactive phosphates, such as hydroxyapatite. The preparation of HAp particles is a factor of crucial importance as the osteoconductivity of the Ti/HAp composite depends on the particle size, particle morphology, and the content of the HAp particles. The development of their specific surface area and the degree of particle agglomeration are determined by drying techniques, synthesis conditions, and the type and concentration of the reagents. Synthesized hydroxyapatite was produced by the wet precipitation method. It was compared to a commercial product through accurate physicochemical studies, confirming the high purity of the obtained synthetic calcium phosphate without any impurities. The next stage of the work focused on developing Ti/HAp composites that are sintered under tailored conditions. The goal of the research was to produce a composite material that resembles the material gradient found in biological materials such as bone. This design helps reduce elastic and compositional mismatches between the implant and bone.

## 2. Materials and Methods

### 2.1. Materials

Hydroxyapatite was synthesized using phosphoric acid (HNO_3_) from Poch Basic (Gliwice, Poland) (CAS no. 7697-37-2), calcium hydroxide (Ca(OH)_2_ from Chempur (Piekary Śląskie, Poland) (CAS no. 1305-62-0), and ammonia water (NH_4_OH, 25%) (CAS no. 1336-21-6) from STANLAB (Lublin, Poland). The composites were prepared using synthesized hydroxyapatite, carboxymethylcellulose, Natriumsalz, M.W. ca.250000 (CAS: 900-32-4), and the titanium alloy (Ti64) Ti-6Al-4V, grade 5, with a particle size range of 15–53 µm, which was purchased from AMC Powders Co., Ltd. (Beijing, China). A reference sheet was an ASTM F 136-13 Wrought Titanium-6Aluminum-4Vanadium ELI) (Extra Low Interstitial) Alloy (R56401) to be used in the manufacture of surgical implants.

### 2.2. Synthesis of Hydroxyapatite

Hydroxyapatite was obtained by the wet precipitation method. This process is characterized by simplicity of execution, low cost, and the possibility of precise control of the reaction parameters. To begin with, precursors—solutions containing calcium cations and phosphate anions—were carefully prepared and mixed in appropriate proportions. During mixing, the pH was controlled, the value of which corresponded to an alkaline environment, which is crucial for the course of the reaction. Under the influence of the appropriate pH and the correct concentration of precursors, calcium and phosphate ions react with each other, leading to the formation of a hydroxyapatite precipitate through a precipitation process. The reaction occurs according to Equation (1):

10 Ca(OH)_2_ + 6 H_3_PO_4_ → Ca_10_(PO_4_)_6_(OH)_2_ + 18 H_2_O (1)

The resulting sludge was then subjected to purification processes—such as filtration and washing to an inert pH and freeze-drying—to obtain a pure final product. During the synthesis, calcium hydroxide of 0.5 mol/dm^3^ and orthophosphoric acid of 0.3 mol/dm^3^ were used as precursors for calcium and phosphorus ions, respectively. The pH value was controlled with the addition of 25% ammonia water.

### 2.3. Preparing Metal/Ceramic Powders

The subsequent step was to prepare a mixture of powders with the specified concentration of hydroxyapatite. Carboxymethylcellulose was used as a binder and was combined with the powder mixture at a mass ratio of either 5% or 10% (Table 1). Two mixtures were prepared: the first consisted of 5% HAp and 5% CMC, while the second contained 10% HAp and 10% CMC. The production of the powders was conducted using a zirconium bowl, zirconium balls, and a planetary mill (PULVERISETTE 6 classic line). The corresponding titanium alloy powder, 5% or 10% HAp and 5% or 10% carboxycellulose, was placed in the zirconium bowl. The ratio of powder to balls was 2:1. In preparing the Ti6Al4V/Hap powder, a grinding speed of 250 rpm was used. The entire process for one mixture was repeated four times, with a 10 min break after each repetition.

### 2.4. Cold Isostatic Pressing

The following step was to subject the powders to a pressing process with an appropriate ceramic content. The Ti6Al4V/HAp powder mixtures were subjected to uniaxial pressing at a force of 15 tons at room temperature. To obtain raw samples with a diameter of 13 mm and a height of 4 mm, an appropriate amount of the mixture was placed in a die. The samples were then dried for 2 h in a laboratory dryer at 300 degrees Celsius.

Two resulting powder mixtures were employed to create a gradient sample. The initial measurement was 0.5 g of a mixture comprising 5% HAp and 5% CMC, which was then placed in a die. Subsequently, 0.5 g of a mixture comprising 10% HAp and 10% CMC was measured and placed in a matrix on top of the mixture of 5% HAp and 5% CMC. Next, the procedure was repeated with an applied pressure of 15 tons.

### 2.5. Sintering Process

**Figure 1:**
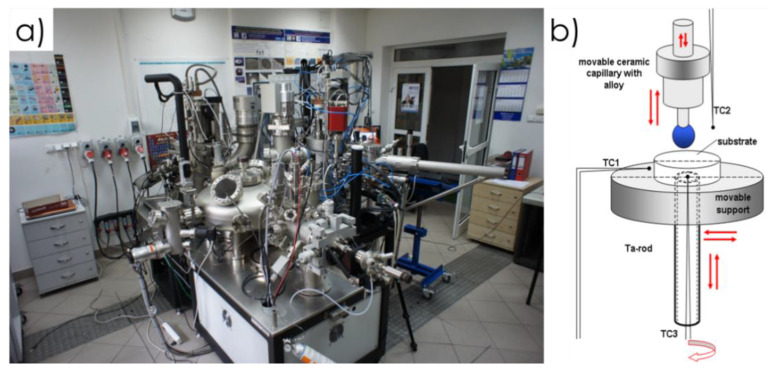
Experimental complex for high-temperature studies (**a**) and the scheme of its movable elements, allowing an examination by different testing methods and procedures (**b**).

The high-temperature sintering process of Ti6Al4V samples with varying HAP and CMC content was conducted using a universal apparatus set designed for high-temperature metal and alloy research (Figure 1) [28], located at the Łukasiewicz Research Network—the Krakow Institute of Technology.

**Figure 2:**
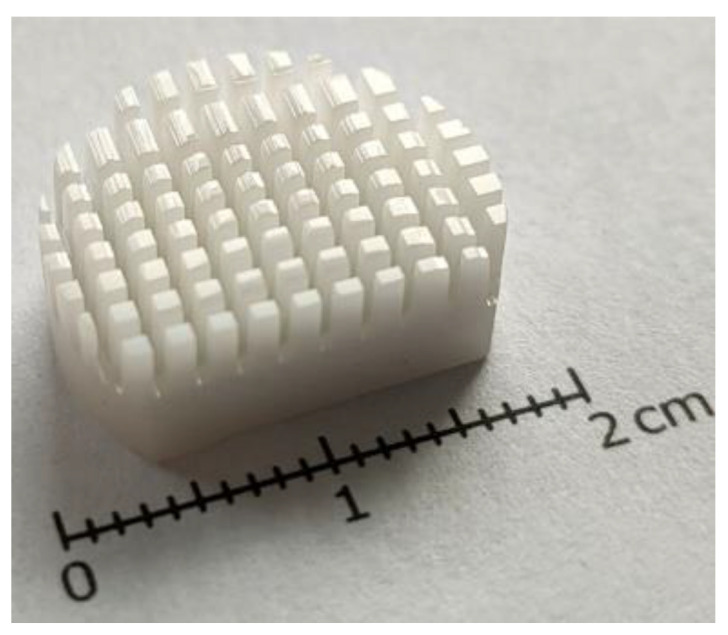
A view of the milled ZrO_2_ substrate.

The experiments were conducted by controlled heating and cooling of Ti6Al4V + HAp + CMC samples placed in the test chamber on specially prepared (milled) substrates made of ZrO_2_ with a diameter of 18 mm and a height of 5 mm (Figure 2), in order to obtain a gradient structure of the composite zones. The milling of the substrates was carried out using the Struers Accutom-100 precise metallographic cutter (Glostrup, Denmark), with the depth of the created channels being 2 mm.

The samples were heated up to *T* = 800 °C (15 °C min^−1^) under a high vacuum of *p* = 10^−7^ mbar; flowing Ar 5.0 (*p* = 850–900 mbar) was introduced into the chamber in order to hinder the evaporation of the Ti6Al4V alloy. The temperature and holding time at this temperature were *T* = 1300 °C and *t* = 30 min, respectively. After this time, the samples were cooled down to room temperature at a rate of 20 °C min^−1^.

**Figure 3:**
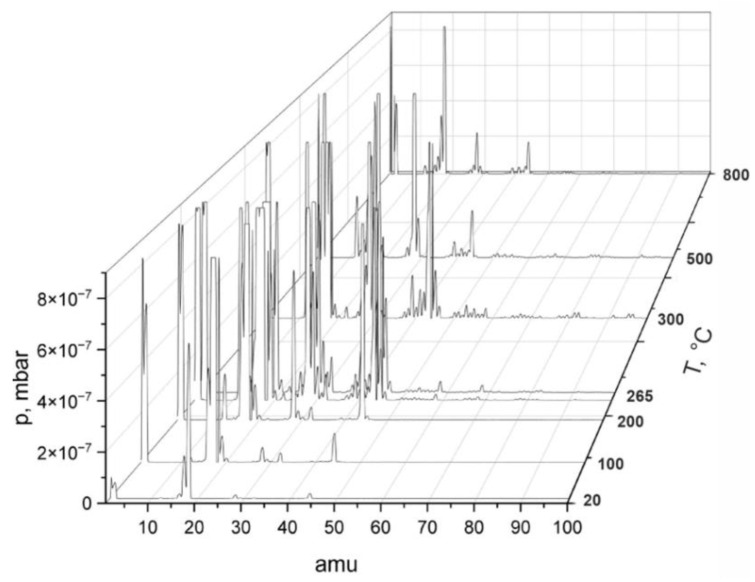
Spectrometry of residual gases in the process chamber during heating up to 800 °C of the Ti6Al4V + 10% HAp + 10% CMC sample.

The sintering of Ti6Al4V + HAp + CMC samples was carried out with continuous recording of residual gas quantities using the Pfeiffer QMS-200 quadrupole mass spectrometer up to a temperature of 800 °C (Asslar, Germany). The recorded changes in the chemical composition of the residual gases during the tests are presented in Figure 3.

The graph presents the strong evaporation of hydrocarbon compounds. This is particularly noticeable after reaching temperatures above 200 °C. Due to the mass of these compounds (amu > 60), it is assumed that they are compounds of organic origin and organic solvents (from 12 to 60 amu).

### 2.6. X-Ray Diffraction 

First, the phase composition of the resulting ceramic powder was checked. For this purpose, X-ray diffraction analysis was carried out. The reference sample was commercial hydroxyapatite (Sigma Aldrich, St. Louis, MO, USA). Table 2 presents the parameters of the XRD analysis performed. The measurement was performed using PANalytical Aeris devices (Malvern PANalytical, Lelyweg, Almelo, The Netherlands).

The degree of the crystallinity of the obtained HAp and commercial material was determined using the crystallinity coefficient (*X*_*c*_), which is defined as the ratio of the intensity of the peaks associated with the crystalline phase to the sum of the intensities of the peaks associated with the crystalline and amorphous phases (according to Equation (2)).

(2) $$X_{c}=\frac{A_{c}}{\left(A_{c}+A_{a}\right)}*100\;\%\;$$

*X_c_*—the degree of crystallinity (%);
*A_c_*—the area under crystalline peaks (*A_c_*);
*A_a_*—the area under amorphous peaks (*A_c_*).

Next, the size of the crystallites, that is, the average size of the crystallite areas in the sample, was determined. For this purpose, the Scherrer equation (Equation (3)) was used.

(3) $$\;B_{k}=\frac{K\lambda }{D_{hkl}cosq}$$

*B_k_*—the reflectance width, dependent on crystallite size, [rad];
*K*—a constant associated with the shape, [-];
*λ*—the radiation wavelength, [Å];
*D*—the size of crystallites in the direction perpendicular to the (hkl), [Å];
*θ*—Bragg’s angle, [°].

### 2.7. Fourier Transform Infrared Spectroscopy

FT-IR analysis was carried out because of its ability to identify the specific chemical bonds of hydroxyapatite, among others. In order to identify the obtained powder, measurements were carried out using a Nicolet iS5 FT-IR spectrometer equipped with an iD7 ATR accessory (Thermo Scientific, Loughborough, UK). The spectra were recorded in the range from 4000 to 400 cm^−1^ (32 scans at 4.0 cm^−1^ resolution) under room conditions.

### 2.8. X-Ray Fluorescence

Fluorescence X-ray analysis of hydroxyapatite was performed to determine the chemical composition of this material, especially the content of elements such as calcium, phosphorus, and any other additives present. A measurement was carried out using an Energy-Dispersive X-ray Fluorescence Spectrometer EDX-7200 (Shimadzu, Kyoto, Japan). The measurement was performed under room conditions.

### 2.9. Particle Size

The particle size distribution was then analyzed. For this purpose, powder samples were analyzed using Anton Paar’s PSA 1190 analyzer (Graz, Austria). The equipment allows the measurement of dry samples in the particle size range from 0.1 µm to 2500 µm. The measurement was repeated three times for each sample.

### 2.10. Determination of the Ca/P Ratio

The determination of calcium content was carried out based on PN-97/R-64803 [29]. In brief, 0.1 g of HAp powder was weighed and placed in 10 mL of 3 M nitric acid (V) solution for 10 min. Then 20 mL of distilled water was added and boiled for the next 5 min. After the solution cooled, it was transferred quantitatively to volumetric flasks, and 6.25 mL of 0.4 M bismuth nitrate solution was added and brought up to the mark with distilled water. The mixed solutions were filtered by discarding the first batch of filtrate. Then 25 mL of filtrate, 25 mL of distilled water, and 3 mL of 25% triethanolamine solution were combined. A total of 10 mL of 20% potassium hydroxide solution and a pinch of indicator were added to the sample. Titration was performed using a standard EDTA solution of 0.2 M. Three replicates were performed. Calcium content was calculated using Formula (4):

(4) $$\%Ca=\frac{0.04008*\;V_{1}*c*\;V_{2}}{m*\;V_{3}}*100$$

*V*_1_—the volume of EDTA solution used during titration;
*c*—titer of EDTA solution;
*V*_2_—the volume of the solution of the test sample;
*m*—the weight of hydroxyapatite sample;
*V*_3_—the volume of solution taken for titration.

Next, the determination of the phosphorus content was carried out in accordance with PN 80/C 87015 [30]. The appropriate hydroxyapatite weights were boiled in the presence of 15 mL of 65% nitric acid (V) solution and 5 mL of 36% hydrochloric acid. After observing a change in the color of the vapor from orange to colorless, the samples were supplemented with 40 mL of distilled water and further boiled. Then, after cooling, they were transferred quantitatively to a flask, made up of distilled water and filtered. A total of 10 mL of the resulting filtrate was combined with 20 mL of solution D and supplemented with distilled water. After 15 min, a measurement was performed using a UV-Vis spectrophotometer ThermoScientific Genesys 180 (Waltham, MA, USA). Three replicates were performed, and the phosphorus content was calculated using Equation (5):

(5) $$\%P=\left(\frac{M_{1}*\;V_{1}*100\%}{m*\;V_{2}*1000}\right)/2.29$$

*M*_1_—P_2_O_5_ content in the analyzed sample determined from the standard curve;
*V*_1_—the volume of the volumetric flask used for phosphorus extraction;
*V*_2_—the volume of solution taken for analysis;
2.29—the conversion factor from P_2_O_5_ to P;
*m*—the weight of hydroxyapatite sample.

### 2.11. Density and Porosity of the Samples

The hydrostatic weighing method (Archimedes method) was used to measure the apparent density of the samples. The density of the sample was determined by weighing the biomaterial in air and after immersion in a liquid of known density (Equation (6)). The sample was initially weighed in air, then transferred to distilled water and weighed again. Once the temperature of the water had been established, the density of the distilled water was determined. Subsequently, the density of the sample was calculated using the following formula:

(6) $$\rho =\frac{m_{1}}{m_{1}-m_{2}}\cdot \rho_{water}$$

*m*_1_—the weight of the dry sample;
*m*_2_—the apparent mass of a sample saturated with liquid and weighed in liquid;
$\rho_{water}$—the density of distilled water at a specific temperature.

Based on the results, calculations were made, and the apparent porosity was determined using Equation (7):

(7) $$P=\frac{m_{1}}{m_{3}-m_{2}}\cdot \rho_{water}$$

*m*_1_—the weight of the dry sample;
*m*_2_—the apparent mass of a sample saturated with liquid and weighed in liquid;
*m*_3_—the mass of a liquid-saturated sample 30 min, weighed in air;
$\rho_{water}$—the density of distilled water at a specific temperature.

### 2.12. Nanomechanical Characterization of the Ti6Al4V/HAp Composites

The nano-compression test was performed using a HYSITRON TS77 nanoindenter (Billerica, MA, USA), which allows for the continuous measurement of elastic modulus and hardness during loading. This is achieved by superimposing a small oscillation on the main load signal and analyzing the resulting system response with a frequency-specific amplifier. In this way, the elastic modulus and hardness can be obtained as a continuous function of penetration.

The penetration was made over a limited area, with a particular focus on the grain. In this instance, a trapezoidal loading cycle was employed, with a maximum load of Pmax = 10,973 μN. The central region of the sintered grain exhibits elevated values of approximately 90 GPa for the reduced elastic modulus and a hardness of 6.11 GPa. The maximum displacement was found to be hmax = 293.1 nm, and the contact stiffness (S) was determined to be 135 µN/nm. In the subsequent phase, nanoindentations were conducted in a grid pattern for the purpose of statistical nanoindentation. To this end, an area of 10 μm × 10 μm was selected on the polished sample surface, and a total of 100 indentations were performed.

### 2.13. The Vickers Microhardness of the Samples

Microhardness is an indicator of the plastic deformation of the manufactured biomaterial. The samples were tested using the Vickers method. The microhardness measurement was performed with a load of 0.5 kg and a magnification of 40×.

### 2.14. Scanning Electron Microscopy with Elemental Composition Analysis

Observations were carried out using scanning electron microscopy to determine the surface morphology of the obtained powder. The samples were dried and then sputtered immediately before measurement. Observations were carried out using JEOL IT200 (JEOL Ltd., Peabody, MA, USA). Prior to measurement, the samples were prepared by sputtering with a gold nanolayer. This was carried out using a Smart Coater DII-29030SCTR (Joel Ltd., Peabody, USA) auto-vacuum sputtering machine.

### 2.15. Optical Microscopy

Surface micrographs and roughness analysis were performed using a Keyence VHX Series Digital Microscope (Osaka, Japan). A high-performance camera allowed us to snap pictures with a total image resolution of 4000 pixels (H) × 3000 pixels (V) with 4 K mode. Observation magnifications were selected at 500 × down to 2500× with the high-resolution HDR function. The measurements of the roughness profile were allowed by applying the 4 K CMOS Sensor of the VHZ-7000 series (Keyence, Osaka, Japan) to perform 2D and 3D measurements.

The roughness profiles were computed from 3D surface measurements, including the parameters Ra (roughness), Rq (kurtosis), and Rsk (skewness), which numerically describe the topography. Ra describes the departures of the roughness profile from the mean line, and Rq is the rms (root mean square) parameter corresponding to Ra. The skewness (Rsk) characterizes the asymmetry of the profile about the mean line, showing a tendency to be in positive or negative values. The mean value and deviation were determined from at least three repetition measurements at different spots on the sample.

## 3. Results

### 3.1. X-Ray Diffraction Analysis

**Figure 4:**
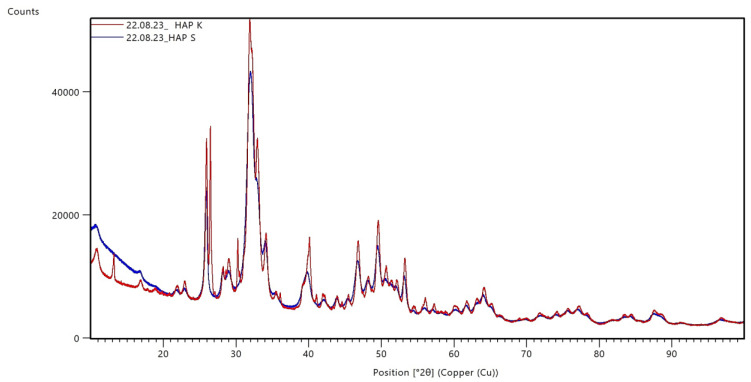
A comparison of commercial (K) and synthetic (S) HAp diffractograms.

The obtained calcium phosphate was analyzed to confirm the phase composition. Diffractograms of the synthetic and commercial powder are summarized in Figure 4.

For both HAp powders, a single phase—hydroxyapatite with the chemical formula Ca_10_(PO_4_)_6_(OH)_2_ and a hexagonal crystal structure—was identified from the ICDD database. It is important to point out that the obtained diffractograms are analogous and consistent with the results of previous researchers [31,32,33]. Moreover, in addition to phase composition, X-ray diffraction (XRD) analysis is a commonly used technique to determine the degree of crystallinity and size of crystallites in crystalline materials such as hydroxyapatite. In the case of HAp, its crystal structure is well-defined and consists of regularly ordered crystallites, making it possible to use XRD to analyze its crystalline features. Thus, X-ray diffraction analysis makes it possible not only to identify the phase and crystal structure of hydroxyapatite but also to assess its degree of crystallinity and crystallite size, which is crucial for understanding and optimizing the properties of this biomaterial. The values of the degree of crystallinity and the crystallite size are summarized in Table 3. For both HAp powders, a single phase—hydroxyapatite with the chemical formula Ca_10_(PO_4_)_6_(OH)_2_ and a hexagonal crystal structure—was identified from the ICDD database. It is important to point out that the obtained diffractograms are analogous and consistent with the results of previous researchers [31,32,33]. Moreover, in addition to phase composition, X-ray diffraction (XRD) analysis is a commonly used technique to determine the degree of crystallinity and the size of crystallites in crystalline materials such as hydroxyapatite. In the case of HAp, its crystal structure is well-defined and consists of regularly ordered crystallites, making it possible to use XRD to analyze its crystalline features. Thus, X-ray diffraction analysis makes it possible not only to identify the phase and crystal structure of hydroxyapatite but also to assess its degree of crystallinity and crystallite size, which is crucial for understanding and optimizing the properties of this biomaterial. The values of the degree of crystallinity and the crystallite size are summarized in Table 3.

Determining the degree of crystallinity and size of hydroxyapatite crystallites is important for several reasons. First, the degree of crystallinity affects the physical and chemical properties of the material, such as its hardness, chemical stability, and surface reactivity. The higher the degree of crystallinity, the better the mechanical and bioactive properties of the material. In addition, the size of the crystallites is important for their bioactivity and reactivity, as smaller crystallites may exhibit a larger specific surface area and greater chemical reactivity [34]. For both parameters, values characteristic of hydroxyapatite powders were obtained, which are not significantly different from those defined for the commercial product.

### 3.2. Fourier Transform Infrared Spectroscopy Analysis

**Figure 5:**
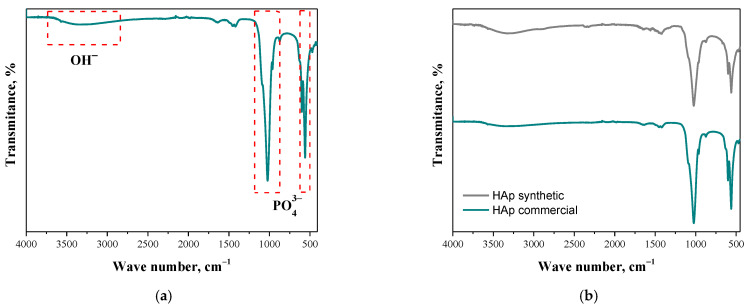
The FT-IR spectrum of commercial HAp (**a**); a comparison of FT-IR spectra of commercial and synthetic HAp (**b**).

Figure 5a presents the FT-IR spectrum of commercial hydroxyapatite, along with the absorption bands assigned, which are characteristic of this material. In turn, Figure 5b shows a comparison of the spectra of commercially and synthetically obtained HAp powders.

Based on the analysis, absorption bands characteristic of hydroxyapatite ceramics were identified. Bands of the particular high intensity characteristic of the phosphate group bond (${\mathrm{PO}}_{4}^{3-}$), occurring at wavelengths of about 560, 600, and 1040 cm^−1^, were identified. Subsequently, the presence of a broad band of low intensity originating from the stretching vibrations of the O-H bond of the hydroxyl group, with a maximum wavelength of about 3400 cm^−1^, was indicated. For both powders, analogous characteristic bands were identified, indicating the high purity of the obtained synthetic calcium phosphate. The characteristic ${\mathrm{PO}}_{4}^{3-}$ and -OH groups in hydroxyapatite have also been confirmed by other researchers [35,36,37]. Based on the analysis, absorption bands characteristic of hydroxyapatite ceramics were identified. Bands of especially high intensity were characteristic of the binding of the phosphate group (${\mathrm{PO}}_{4}^{3-}$) and a broad band of low intensity originating from the stretching vibrations of the O-H bond of the hydroxyl group. For both powders, analogous characteristic bands were identified, indicating the high purity of the obtained synthetic calcium phosphate. The characteristic ${\mathrm{PO}}_{4}^{3-}$ and -OH groups in hydroxyapatite have also been confirmed by other researchers [35,36,37].

### 3.3. X-Ray Fluorescence Analysis

**Figure 6:**
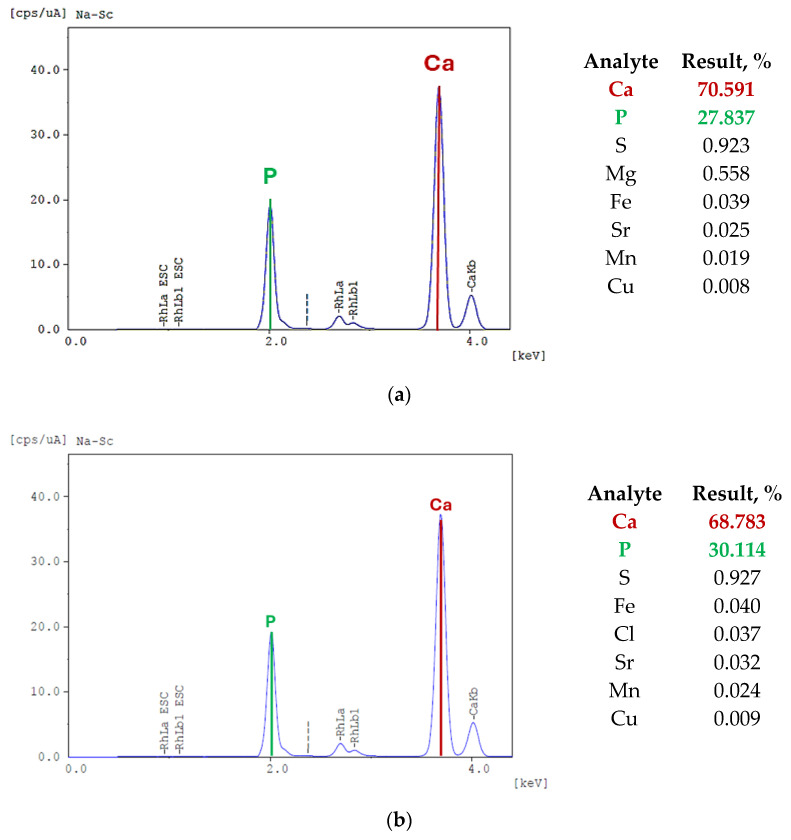
Results of the XRF analysis of synthetic HAp (**a**) and commercial HAp (**b**).

Next, XFR analysis was performed, with results providing information on the elemental composition of the analyzed sample. The results are presented in Figure 6.

The XRF analysis of the hydroxyapatite showed that the dominant elements in the sample are calcium (70.591%) and phosphorus (27.837%), which is as expected for pure hydroxyapatite, as these two elements are its main components. The presence of other elements, such as sulfur (0.923%), magnesium (0.558%), iron (0.039%), strontium (0.025%), manganese (0.019%), and copper (0.008%), can be disregarded, as they do not significantly affect the overall properties of the material. The total amount of other elements is about 1.5% and can come from laboratory apparatus, instruments, and laboratory air. Even trace contaminants in chemicals and auxiliary materials can contribute to the presence of these elements in a sensitive XRF method. However, large amounts of elements such as calcium and phosphorus are crucial in this analysis. Similar results were obtained for both powders.

XRF measurements were also conducted for the samples after sintering (Table 4), and the tests confirmed the presence of all constituent elements. The composition also shows the elements calcium and phosphorus, which confirms the presence of calcium phosphates after the sintering process. The measurement does not penetrate deep into the sample, which is why the values are low; moreover, chipping from the surface must be taken into account in the case of sintering in a vapor phase.

### 3.4. Particle Size Analysis

**Figure 7:**
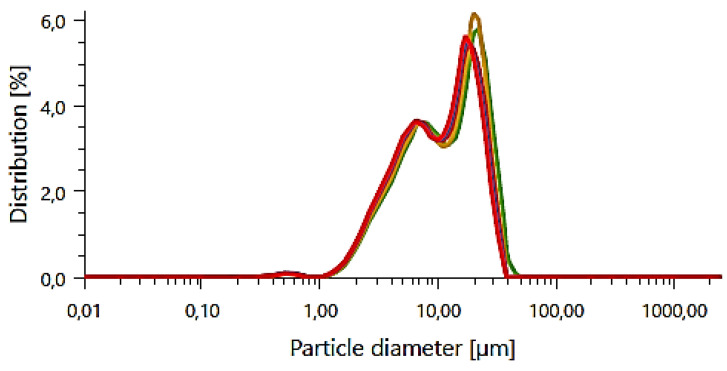
The particle size distribution of the obtained HAp (each colored line corresponds to one of the 3 repetitions).

The analysis of the hydroxyapatite particle size was aimed at determining the precise particle size distribution in the sample. This provides detailed information on the particle size distribution of hydroxyapatite, allowing for the optimization of the manufacturing process and tailoring of material properties for specific applications in medicine, biomaterial engineering, and other fields. The results of the PSA analysis are presented below in Figure 7 and Table 5 and Table 6.

The results of the synthetic hydroxyapatite particle size analysis showed an average particle size of about 12 μm. Adequate particle size is important because it affects the interaction between the implant and surrounding tissues and the regeneration processes of bone tissue. The resulting HAp with a relatively homogeneous and low particle size should ensure adequate tissue penetration and interaction between the implant and bone, which is crucial for the integration of the implant into the host tissue. In addition, this particle size provides an adequate surface area for protein and cell adsorption, which can promote bone cell adhesion and regeneration processes. Micro-sized hydroxyapatite similar to the results presented above was also studied by Liu. He conducted in vivo studies using nano-sized HAp, micro-sized Hap, and a combination of both. It was proved that HAp implantation after 28 days was successful for both nano- and micro-HAp. These results indicate the high potential of the resulting ceramic powder [38]. Hydroxyapatite has been extensively studied in biomedical applications at both the nano- and micro-size. For example, Lv et al. tested the efficacy of combining nano-hydroxyapatite with vancomycin in the treatment of chronic osteoarthritis with bone defects. The study showed that a composite containing PLA and nano-HAp with vancomycin effectively prolongs the antibiotic’s duration of action and better penetrates the bone marrow [39]. On the other hand, in the presented work, hydroxyapatite shows its size to be in the micrometric range, similar to the work of Liu et al. Liu et al. conducted in vivo studies using nano-sized HAp, mirco-sized Hap, and a combination of both. It was proved that HAp implantation after 28 days was successful for both nano- and micro-HAp. These results indicate the high potential of the resulting ceramic powder [38]. It is also worth noting that similar results were obtained by Kumar et al., who focused on the development of composite biomaterials based on titanium and micro-hydroxyapatite. Their mechanical studies demonstrated that such a combination minimizes the impact of stress shielding due to the altered structure of the composite containing micro-HAp compared to pure metal [40].

### 3.5. Scanning Electron Microscopy with Elemental Composition

SEM analysis with EDS elemental composition for HAp powder was conducted to understand its morphology, structure, and chemical composition. SEM allows high-resolution images of the powder’s surface to assess its morphology and micro- and macromolecular structure. This analysis also makes it possible to assess the purity and homogeneity of the powder, which is important for its quality and consistency, especially in biomedical applications, as these parameters are crucial for the safety and effectiveness of biomaterials.

**Figure 8:**
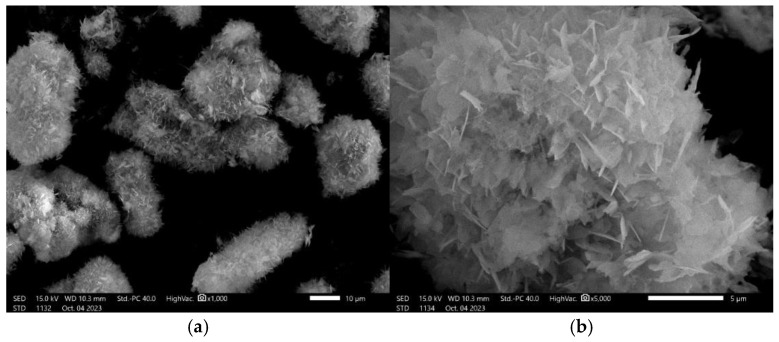
Scanning electron microscopy images at magnification ×1000 (**a**) and ×4000 (**b**).

Figure 8 presents SEM images of the ceramic powder at various magnifications. During the analysis, it was confirmed that the obtained hydroxyapatite has a relatively high homogeneity. These results are consistent with the PSA analysis results presented above. Particularly important is the morphology and surface topography of the obtained material. As can be seen in Figure 8b, the obtained HAp is characterized by a complex surface morphology, which may be particularly important in biomedical applications. Surface roughness enhances cell adhesion, proliferation, and differentiation, especially of osteoblasts, which are key to bone tissue regeneration. Additionally, the increased surface area resulting from the roughness improves protein adsorption, promoting better biological interactions and accelerating the process of osseointegration [41]. The study conducted by Deng et al. demonstrated a significant correlation between surface roughness and bone cell proliferation. The results showed that the increased surface roughness of the composite with hydroxyapatite led to a substantial improvement in hydrophilicity and an increase in the amount of calcium ions on the surface, which promoted better adhesion and the proliferation of MG-63 osteoblast-like cells. Notably, composites with moderate surface roughness significantly enhanced cell attachment and proliferation and promoted alkaline phosphatase (ALP) activity and calcium nodule formation, compared to other groups, indicating markedly improved osteogenic properties [42].

**Figure 9:**
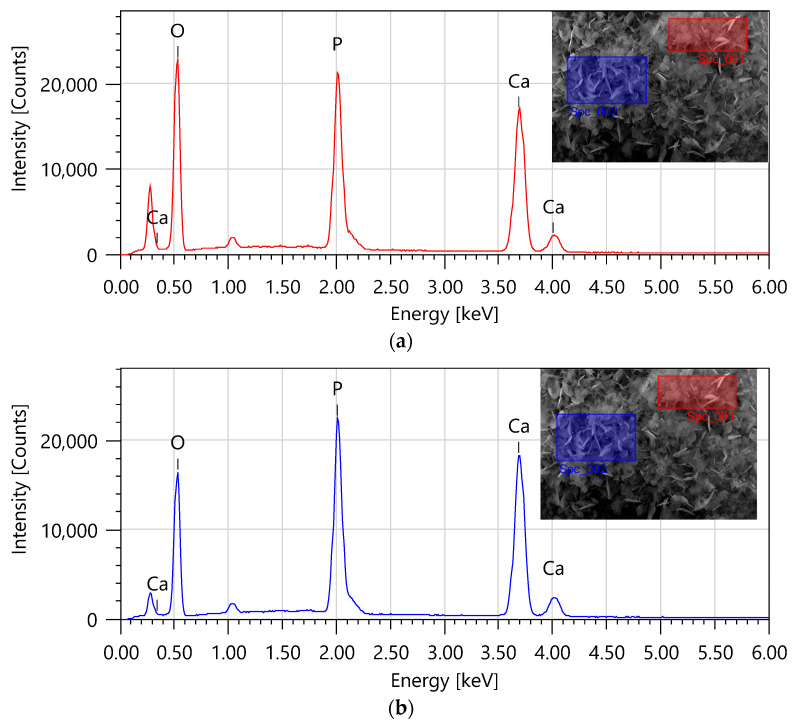
Elemental composition analysis from the area marked with a red box (**a**) and a blue box (**b**).

The chemical composition of the resulting HAp powder was determined. Analysis was carried out at two locations. The results are presented in Figure 9a,b. In both cases, a significant content of calcium and phosphorus was identified, which confirms the presence of elements characteristic of hydroxyapatite and is consistent with the results presented above.

**Figure 10:**
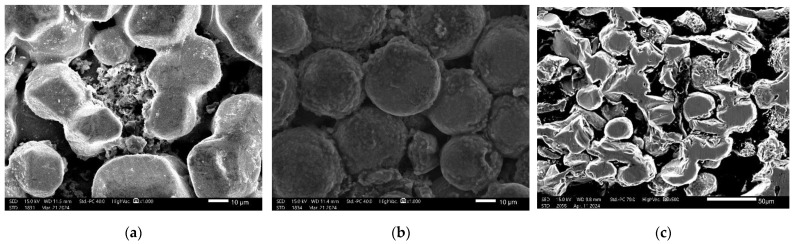
Morphology analysis of the samples after sintering at magnification ×1000 for (**a**) 5%Hap5%cmc and (**b**) 10%Hap10%cmc (**c**) and the cross-section of the gradient structure at magnification ×500.

To investigate the surface of the fabricated composites, scanning electron microscope analysis and energy-dispersive X-ray spectroscopy (EDS) analysis were conducted on the sintered samples. The SEM images are presented in Figure 10 The EDS analyses confirm that the elemental composition of each of the fabricated biomaterials is accurate with respect to titanium (Ti), aluminum (Al), vanadium (V), calcium (Ca), phosphorus (P), and oxygen (O). The presence of specific elements, such as aluminum (Al) and vanadium (V), is directly correlated with the specific titanium alloy utilized in the fabrication of Ti6Al4V/HAp biomaterials. It is also worth noting that the presence of elements such as calcium (Ca) and phosphorus (P) is associated with the content of hydroxyapatite. The introduction of this element into the system is to introduce properties such as bioactivity.

**Figure 11:**
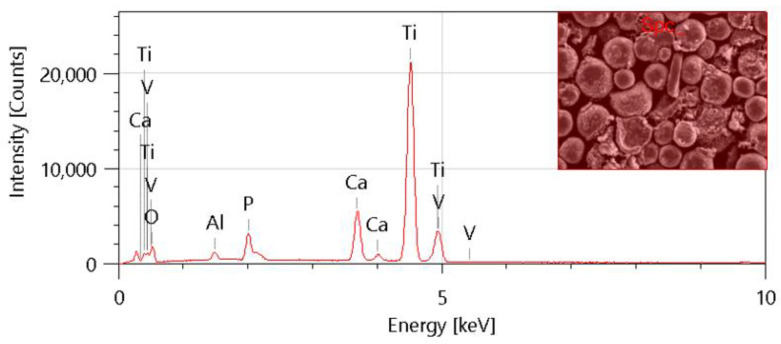
Elemental composition analysis for sample after sintering 10%Hap10%CMC.

The EDS analysis corroborates the presence of elevated hydroxyapatite concentrations in the samples containing 10% Hap (Figure 11).

The results of the scanning electron microscopy (SEM) analysis after sintering indicate the presence of uniform titanium grains with a spheroidal morphology as well as the dispersion of hydroxyapatite between these grains. SEM images indicate the absence of cracks in the material and the absence of other notable alterations. The analysis has demonstrated that the synthesized material is indeed porous in nature. The data indicated that materials with a higher content of porophore exhibited a greater number of larger pores. The presence of pores with a diameter of 10–50 µm has been identified as a factor that facilitates the diffusion of cells into the material.

**Figure 12:**
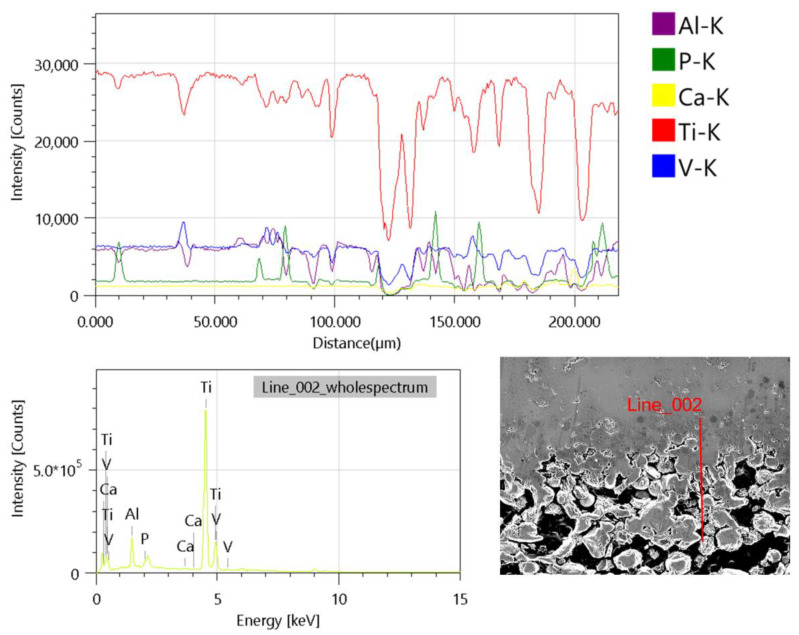
Line Scan Profile for gradient sample.

The results of the scanning electron microscopy (SEM) analysis corroborate the presence of a gradient structure within the material, which is indicative of a porous zone and a compacted zone. The porous structure corresponds to 5% Hap5%cmc, while the compact structure is 10%Hap10%cmc. There is a uniform distribution of 10–100 μm pores within the porous structure. A pore size of approximately 50 μm is very important for bone regeneration implants. Pores of this size allow for osteointegration and bone ingrowth into the implant [43]. The presence of all elements in the composite is also confirmed by linear EDS measurements of a gradient sample (Figure 12) 

### 3.6. Determination of Ca/P Ratio

Analysis of the Ca/P ratio in hydroxyapatite is crucial, because the ratio determines the type and properties of the calcium phosphates formed. The ideal Ca/P ratio for stoichiometric hydroxyapatite is about 1.67, which corresponds to its chemical formula Ca_10_(PO_4_)_6_(OH)_2_. Deviations from this ratio may indicate the presence of other forms of calcium phosphates, such as tricalcium phosphate (Ca/P = 1.5) or brushite (Ca/P = 1.0). Each of these forms has different physicochemical and biological properties that affect the biocompatibility, resorption, and mechanical properties of the material. Therefore, the precise determination of the Ca/P ratio is essential for understanding and controlling the quality and function of phosphate materials used in medicine and biology.

The analysis of the Ca/P ratio for the obtained HAp powder showed a value of 1.68, which is very close to the ratio of 1.67, characteristic of stoichiometric hydroxyapatite (Table 7). Such a result indicates the high purity and quality of the material, confirming that the sample is hydroxyapatite with the correct chemical composition. In addition, it can be concluded that the material has the correct proportions of calcium and phosphorus, which is crucial for its optimal biological and physicochemical properties.

### 3.7. Density and Porosity

The results of the densities of the samples used are similar (Table 8). The sample with 5% Hap and 5% carboxymethylcellulose has a higher density—4.434 g/cm^3^. The sample containing 10% Hap and 10% carboxymethylcellulose has a slightly lower density—4.250 g/cm^3^. Hydroxyapatite has a density of approximately 3.1 g/cm^3^. In contrast, the density of the titanium alloy Ti6Al4V used to fabricate the samples is 4.43 g/cm^3^. The difference in the density of the samples tested is due to the difference in hydroxyapatite content. The higher content of hydroxyapatite contributes to the lower density of the biomaterial. From the calculated data, less porogen provides less porosity. The assumption of the addition of carboxymethylcellulose does not coincide with the calculated porosity, and this is due to the fact that the method involves a high degree of inaccuracy, based on a large measurement error. The spalling of the material during drying and sintering must also be taken into account. The carboxymethylcellulose content was removed during the sintering process at around 200–300 °C, which may have led to the simultaneous removal of material adjacent to the CMC. This may be the reason for the formation of higher porosity than assumed.

### 3.8. Nanointender Analysis

**Figure 13:**
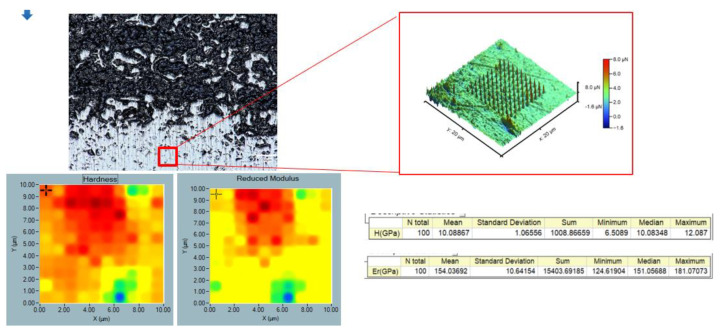
Nanointender analysis for gradient sample after sintering.

The static nanoindentation test was performed on the polished surface of the gradient sample, where Young’s modulus was found to be 154 ± 10.6 GPa (Figure 13). For a comparison, the Young’s modulus of the reference Ti6Al4V titanium alloy polished sheet is about 114 GPa. This value is close to the Young’s modulus of the gradient specimen. Based on the results, it can be concluded that the test specimen is more resistant to deformation and undergoes less deformation under the influence of a given force compared to the titanium alloy specimen. Ti6Al4V titanium alloy has a Young’s modulus that deviates from that of bone, ranging from 10 to 30 GPa. However, compared to medical steel and Co-Cr-Mo alloys used in biomedical applications, the Young’s modulus of Ti6Al4V is half that [43]. Moreover, Young’s modulus and the hardness were measured on the non-porous part. It is impossible to perform the presented analysis on the porous part. The result that is presented relates to the analysis on the non-porous part, hence the Young’s modulus values are much higher than the Young’s modulus of bone. The static nanoindentation test has shown that the nanohardness of the tested gradient sample is 10 ± 1 GPa [43].

### 3.9. Microhardness Analysis

A hardness test was conducted on the compacted region of the gradient sample and subsequently compared with that of a reference sample, which was a sheet of pure titanium alloy Ti6Al4V. The reference is a titanium sheet because it is the basic material for metal implants. Table 9 summarizes the results. For each sample, three measurements were taken at different locations, and the average was calculated. Tests to measure the hardness of the gradient sample indicated a value close to that of the reference sample. The analysis revealed that the hardness values for the pure titanium alloy Ti6Al4V were slightly higher. Additionally, the Ti matrix can be associated with the addition of a ceramic phase and the porous structure of the gradient sample, which results in a slight reduction in the mechanical strength of the sample.

### 3.10. Optical Microscopy Analysis

#### 3.10.1. Composites Before Sintering

**Figure 14:**
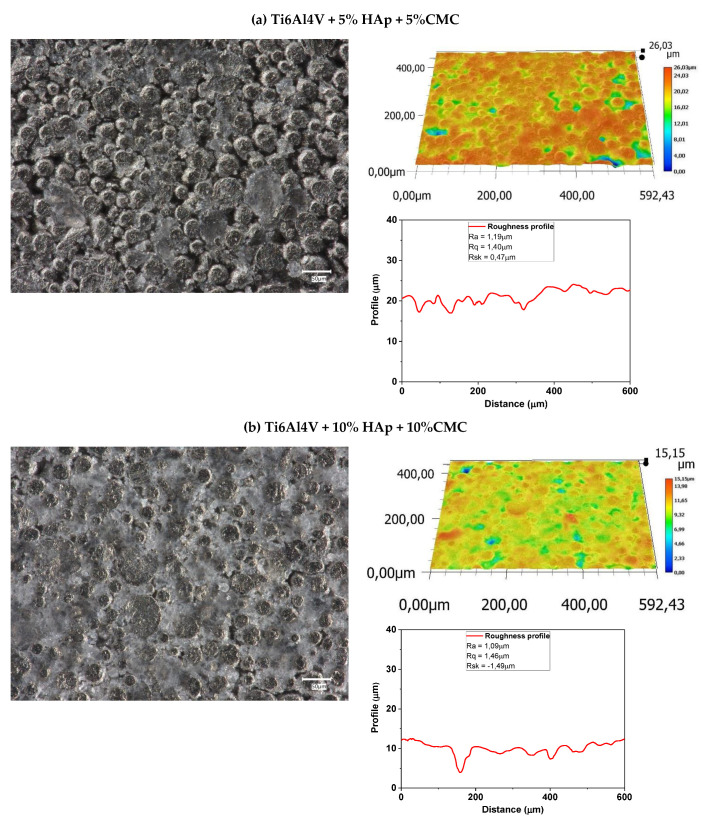
The optical microscopy analysis and roughness profile for samples before sintering: (**a**) Ti6Al4V + 5% HAp + 5%CMC and (**b**) Ti6Al4V + 10% HAp + 10%CMC.

The analysis of the surfaces before the sintering process, visible in Figure 14, shows smooth surfaces along with corresponding profiles representing a low Ra roughness parameter oscillating around 1 µm and an Rq root mean square around 1.4 µm. These results are attributed to the fact that the porosity is filled in with the loose powder of HAp and CMC. Loose powder is visible on the micrographs as light grey cauliflower patches, which fill all the cavities and defects in the CIP-pressed composites.

#### 3.10.2. Composites After Sintering

**Figure 15:**
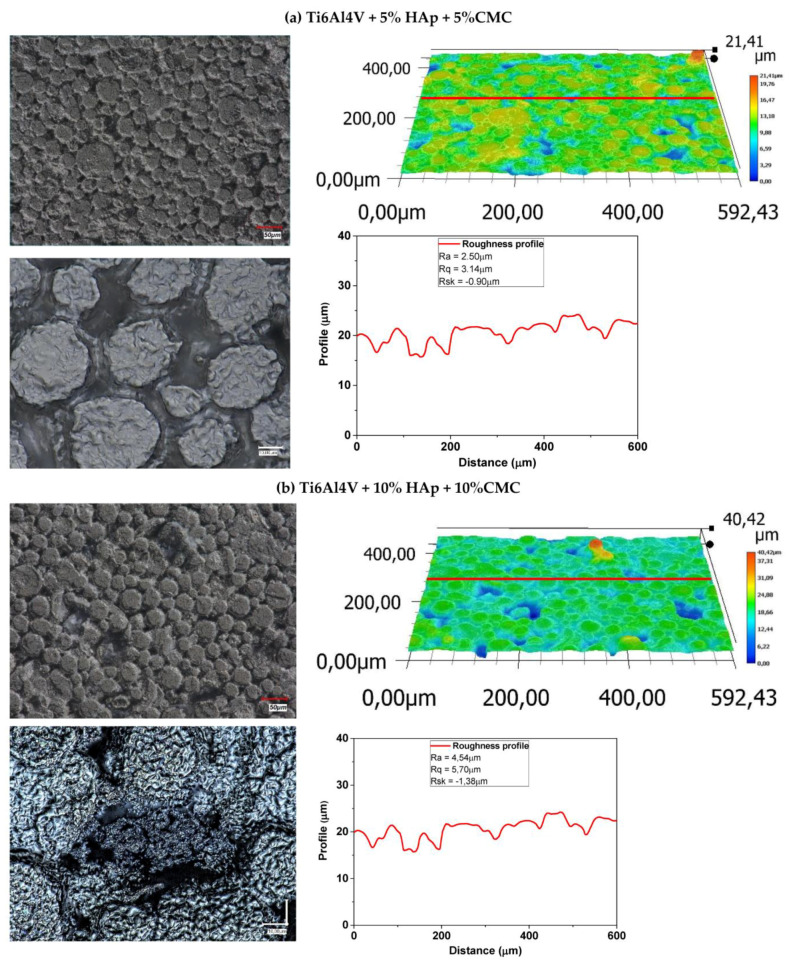
The optical microscopy analysis and roughness profile for the sample after sintering: (**a**) Ti6Al4V + 5% HAp + 5%CMC and (**b**) Ti6Al4V + 10% HAp + 10%CMC.

The surfaces after sintering are more compact compared to the surfaces before sintering. The light grey patches cannot be detected anymore, which is attributed to CMC (porophore) completely evaporating them in the vacuum during the sintering process, while HAp is only visible inside the cavities and the pores, which are left after CMC (Figure 15).

**Figure 16:**
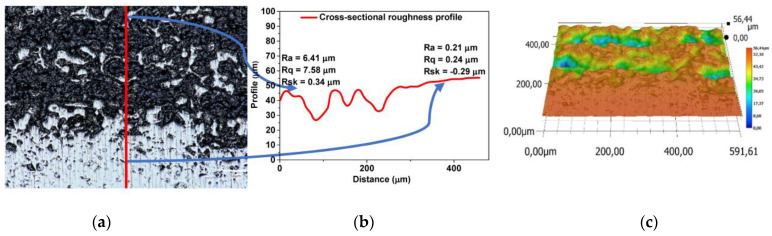
Optical Microscopy analysis for the gradient sample after sintering: (**a**) cross-sectional micrograph, (**b**) roughness profile, and (**c**) 3D image.

The cross-sectional micrograph of the gradient sample is presented in Figure 16a, together with the cross-section roughness profile (Figure 16b) and 3D image (Figure 16c). The lower part of the sample is smooth sintered Ti6Al4V with few pores. During sintering, the ZrO_2_ plate was laying just underneath, causing a longer temperature transfer to the Ti/HAp composite, resulting in a gradient structure. The upper part of the cross-section micrograph is the Ti/HAp composite, where the black spots are the empty pores (after CMC), the blueish spots are the Hap, and the light grey spots represent Ti6Al4V. Clearly, the roughness is much higher in the composite part (Ra = 6.41 µm) compared to the smooth sintered Ti6Al4V part, where the roughness oscillates around 0.2 µm.

## 4. Discussion

Studies show that hydroxyapatite can have a stimulating effect on bone reconstruction. It has the ability to resorb in the human body, which makes it often used for bone tissue reconstruction. Due to its properties, such as its biocompatibility, it is often applied as a coating on metallic materials. The weak adhesion between the two phases, i.e., titanium alloy Ti6Al4V and hydroxyapatite, means that producing composites containing these two phases using the powder metallurgy (PM) method provides excellent mechanical properties without a mechanically weak interface. The precise selection of pressing and subsequent sintering parameters, as well as the appropriate selection of the porophore, produces an implant that should provide a good supply of body fluids to the developing bone cells, which in turn will accelerate the bone reconstruction process. In addition, the creation of a gradient material from two phases, with different ceramic contents, allows for the reproduction of the bone structure and will contribute to the reduction in elastic mismatch.

Over the years, many people have attempted to produce implants based on titanium alloys and hydroxyapatite. However, much of the research that has been completed involves titanium alloy coated with hydroxyapatite. In this situation, however, there is a risk of poor adhesion of the manufactured coating. Studies of this type are presented in the following article: “Biomimetic Deposition of Hydroxyapatite Layer on Titanium Alloys” by Madalina Simona Baltatu et al. [44].

Introducing HAp inside the composite seems to be a better option that can lead to bone regeneration in a more efficient manner. In one case, at least, we nullified the risk of poor adhesion between the two phases (Gurpreet Singh et al. [44]).

In their paper, Gurpreet Singh et al. also attempted to produce a Ti6Al4V/Hap-based composite using the powder metallurgy method. They fabricated several samples that varied in ceramic content and studied the effect of temperature on the mechanical properties of the biomaterials. Our work, while also describing the same components, points to an innovative design of a gradient structure created from two compositions. Moreover, the addition of CMC allows the introduction of pores of the appropriate size.

There are many other material combinations, such as metal-polymer, that also hold promise as implantable materials. Applied by A.V. Okulov and others [45], BPF polymer is a promising polymer with strong antioxidant properties. However, the polymer is not a natural component of bone-like hydroxyapatite, so for bone implants, the addition of ceramics may be crucial.

Conducted studies have shown that the Ti/HAp composite exhibits a favorable gradient structure with optimal porosity and mechanical properties, making it suitable for bone regeneration applications. SEM studies confirm the presence of homogeneous titanium grains with spheroidal morphology and the dispersion of hydroxyapatite between these grains. The analysis confirms the porosity of the material, which increases with higher amounts of added porophore. The pores in the created structure with a size of 10–100 μm are an important element of the bone implant. They allow for osteointegration and bone cell growth. Another crucial element during biomaterial fabrication is hydroxyapatite and its Ca/P ratio. Its analysis allowed us to determine the Ca/P ratio and, at the same time, the presence of calcium phosphate types. In the case of stoichiometric hydroxyapatite, the ratio is 1.67, and deviations from this ratio indicate the presence of other calcium phosphates, such as tricalcium phosphate (Ca/P = 1.5) or brussite (Ca/P = 1.0). Furthermore, the extensive physico-chemical analysis of the hydroxyapatite shows its compatibility with literature data and confirms the selection of the correct manufacturing method. XRF analysis of the hydroxyapatite confirms the presence of all selected elements. An important aspect of biomaterial development is its mechanical properties, including the microhardness and Young’s modulus. A reference sheet of titanium alloy Ti6Al4V shows a high Young’s modulus of 114GPa. Similar values characterize the tested gradient sample. Based on the results, it can be concluded that the test sample is more resistant to deformation and undergoes less deformation under a given force compared to the titanium alloy sample. The value of Young’s modulus, however, remains high and deviates from the Young’s modulus of bone. It should be noted, however, that compared to other materials used in biomedicine, this value is half as high.

By selecting the correct sintering parameters in a high vacuum, the successful integration of hydroxyapatite into the titanium alloy was achieved. The evaporation of residual gases from CMC was particularly noticeable in the temperature range of 200–300 °C, reaching stability above. Scanning microscopy analysis confirmed the feasibility of forming the gradient structure in our proposed manner and proved the porosity to allow cell penetration. The presence of pores with a size of 10–100 μm was confirmed, which is seen as an opportunity for bone cell penetration and thus bone growth. The gradient structure is designed to simulate the structure of bone, i.e., a compact and spongy structure. The better fit of the implant to the bone tissue presumably should guarantee a better fit and faster bone regeneration. An XRF examination of the samples after sintering confirmed the presence of crystallinity phases from hydroxyapatite. The lower calcium and phosphorus content is related to the fact that during the test the measurement does not reach deep into the sample, where the hydroxyapatite content should be the highest.

The achieved gradient Ti6Al4V alloy with bioactive HAp porous structures strongly increases osteointegration potential, thus confirming the suitability of this biomaterial for bone regeneration.

## 5. Conclusions

In the present study, the physicochemical characterization of hydroxyapatite and Ti6Al4V/HAp composite was carried out, and the following conclusions were drawn:

1. The synthesized hydroxyapatite was successfully produced by the wet precipitation method, as confirmed by physicochemical studies. Its properties were compared with commercial hydroxyapatite.
2. Ti6Al4V/HAp composite was prepared by powder metallurgy (PM) method and then sintered in a vacuum.
3. An innovative gradient structure consisting of two compositions (5%HAp%5CMC and 10%HAp10%CMC) was developed to mimic the structure of bone tissue.
4. Pores of 10–100 µm in size were observed, which, when placed in the human body, will enable bone cells to penetrate the implant and regenerate bone.
5. The addition of hydroxyapatite was observed to reduce the microhardness of the composite.

The presented results represent the biomedical potential of the fabricated gradient composite based on Ti6Al4V titanium alloy and HAp.

Further research will focus on the incubation of manufactured materials in fluids simulating the environment of the human body and biological studies of cell penetration into porosity.

The high-temperature sintering process of Ti6Al4V samples with varying HAP and CMC content was conducted using a universal apparatus set designed for high-temperature metal and alloy research [28], located at the Łukasiewicz Research Network—Krakow Institute of Technology.

## Figures and Tables

**Table 1:** Sample composition.

	Sample	Titanium Alloy	Hydroxyapatite [Hap]	Carboxymethylcellulose [CMC]
1.	5%Hap5%CMC	Ti6Al4V	5%	5%
2.	10%Hap10%CMC		10%	10%
3.	5%Hap5%CMC/10%Hap10%CMC Gradient sample		5% first layer 10% second layer	5% first layer 10% second layer

**Table 2:** X-Ray analysis parameters.

Parameter	Value
Angle range	9.999–100 °2θ
Measurement step	0.0027166 °2θ
Counting time	340,425 s
Total time per measurement	13 h 2 min 32 s
Components	Nickel filter on the lamp Mask 13 mm Gap 1° Knife in position LOW

**Table 3:** Crystallinity parameters of HAp powders.

Parameter	Synthetic HAp	Commercial HAp
Crystallite size [nm]	11.38	18.25
Degree of crystallinity [%]	58.97	70.93

**Table 4:** Results of the XRF analysis samples after sintering.

Sample	Analyte	Result [%]
5%Hap5%CMC	Ti	91.501
	Al	3.026
	V	2.638
	P	2.293
	Ca	0.191
10%Hap10%CMC	Ti	89.262
	P	4.325
	V	3.659
	Al	1.265
	Ca	1.249

**Table 5:** Details of PSA analysis results for synthetic HAp.

	D_10_ [µm]	D_50_ [µm]	D_90_ [µm]	Mean Size [µm]
Mean value	3.097	9.951	23.137	12.466
Standard deviation	0.09694	0.5536	1.5211	0.7865
Rel. Standard deviation	3.13	5.56	6.57	6.13

**Table 6:** Details of PSA analysis results for commercial HAp.

	D_10_ [µm]	D_50_ [µm]	D_90_ [µm]	Mean Size [µm]
Mean value	2.162	6.890	15.121	8.294
Standard deviation	0.04529	0.2283	0.4845	0.2681
Rel. Standard deviation	2.09	3.31	3.20	3.23

**Table 7:** Analysis of the Ca/P ratio (%Ca—percentage of calcium, n Ca—molar content of calcium, % P—percentage of phosphorus, n P—molar content of phosphorus, Ca/P—molar ratio of calcium and phosphorus).

Repetition	%Ca	n Ca	%P	n P	Ca/P
1	36.4728	0.910045	16.88066	0.544995	1.669825
2	38.8776	0.970048	17.57588	0.56744	1.709518
3	38.076	0.950047	17.61933	0.568842	1.670141
Average Ca/P ratio					1.683161

**Table 8:** The porosity and density of the samples.

Sample	Density of Sample [g/cm^3^]	Porosity [%]
5%Hap5%cmc	4.434	25
10%Hap10%cmc	4.250	35

**Table 9:** Microhardness Vickers analysis.

Sample	1 [HV]	2 [HV]	3 [HV]	Average [HV]
Gradient sample	351	366	411	376
Ti6Al4V Sheet	410	416	420	415

## Data Availability

The original contributions presented in the study are included in the article, further inquiries can be directed to the corresponding authors
